# IPDfromKM: reconstruct individual patient data from published Kaplan-Meier survival curves

**DOI:** 10.1186/s12874-021-01308-8

**Published:** 2021-06-01

**Authors:** Na Liu, Yanhong Zhou, J. Jack Lee

**Affiliations:** Department of Biostatistics, The University of Texas, MD Anderson Cancer Center, Houston, United States

**Keywords:** Individual patient data (IPD), Kaplan-Meier curve, Meta-analysis, *R* package, Shiny application, Survival analysis

## Abstract

**Background:**

When applying secondary analysis on published survival data, it is critical to obtain each patient’s raw data, because the individual patient data (IPD) approach has been considered as the gold standard of data analysis. However, researchers often lack access to IPD. We aim to propose a straightforward and robust approach to obtain IPD from published survival curves with a user-friendly software platform.

**Results:**

Improving upon existing methods, we propose an easy-to-use, two-stage approach to reconstruct IPD from published Kaplan-Meier (K-M) curves. Stage 1 extracts raw data coordinates and Stage 2 reconstructs IPD using the proposed method. To facilitate the use of the proposed method, we developed the *R* package *IPDfromKM* and an accompanying web-based Shiny application. Both the *R* package and Shiny application have an “all-in-one” feature such that users can use them to extract raw data coordinates from published K-M curves, reconstruct IPD from the extracted data coordinates, visualize the reconstructed IPD, assess the accuracy of the reconstruction, and perform secondary analysis on the basis of the reconstructed IPD. We illustrate the use of the *R* package and the Shiny application with K-M curves from published studies. Extensive simulations and real-world data applications demonstrate that the proposed method has high accuracy and great reliability in estimating the number of events, number of patients at risk, survival probabilities, median survival times, and hazard ratios.

**Conclusions:**

*IPDfromKM* has great flexibility and accuracy to reconstruct IPD from published K-M curves with different shapes. We believe that the *R* package and the Shiny application will greatly facilitate the potential use of quality IPD and advance the use of secondary data to facilitate informed decision making in medical research.

## Background

Typical information used for meta-analysis of survival data reported from clinical trials often includes a summary of outcomes for each arm, including but not limited to hazard ratios and Kaplan-Meier (K-M) curves along with the number of patients at risk [1]. When applying secondary analysis on such published survival data, difficulties usually come from insufficient details in the reported data, which are often reported using aggregated summary statistics. For example, when conducting the meta-analysis on time-to-event data, it is possible that the proportional hazard ratio assumption may not hold, and alternative measures of the survival difference are needed to avoid bias [2, 3]. In this case, it is of great importance to obtain individual patient data (IPD), with which one can not only perform the standard survival analysis, but also can assess if the assumption of the original method is appropriate in order to decide whether or not alternative methods should be applied [4]. Furthermore, one can undertake additional subgroup analyses not reported in the aggregated data. For this reason, the IPD approach is considered as the gold standard in data analysis.

However, researchers conducting meta-analysis or other secondary analyses may lack access to the IPD, partly due to the confidentiality of clinical data. Therefore, a method that is able to reconstruct IPD from published K-M curves can greatly facilitate secondary analyses on survival data. Several methods have been reported in the literature. The iterative algorithm based on K-M estimation method (referred to as “iKM” hereafter) proposed by Guyot et al. [5] is a classic approach among many proposed. It has been used in various secondary analyses. For example, Satagopan et al. [6] used it to reconstruct time-to-event data from a melanoma data set for evaluating different treatment benefits according to biomarker subgroups. Wei and Royston [7] developed a STATA function to apply the iKM algorithm with some adaptions and applied it to reconstruct IPD from K-M curves from multiple trials.

However, there are a few factors that limit the use of the iKM method. First, external software is needed to extract data points before using the iKM algorithm. The original iKM method suggests a manual approach to pick up a sufficient number of points from K-M curves via mouse-clicks using the DigitizeIt software.

Second, the iKM method has restricted requirements when picking up the points manually: (1) the survival probability needed to decrease monotonically as time increases, (2) the points where the number of patients at risk are reported must be included, and (3) users need to sort the data coordinates into time intervals determined by the time points, at which the number of patients at risk is reported. K-M curves in publication typically report the number of patients at risk at several time points under the x-axis. If the number of patients at risk is reported at month 3, 6, and 9, then data coordinates need to be manually organized into the time intervals: [0−3),[3−6),[6−9), and [9,+*∞*). The first two requirements are generally hard to keep for a large amount of manual mouse-clicks and the last one is also time consuming. Thus, it is important to have a flexible function to perform these tasks automatically.

Third, there is a numeric issue in the original iKM method: estimation can be negative values in certain scenarios. Our examination reveals that this is due to a boundary setup in the iterative process.

Finally, there is a lack of user-friendly software for clinical researchers to use for this method. The published functions [5, 6, 8] for the iKM method have at least one of the following inconveniences: external digitizing software is need for data extraction; users need to manually check if data input is appropriate, or the auto process program is not convenient or stable; or no accuracy assessment is provided for users to directly evaluate the reconstruction results within the same software.

To overcome the aforementioned limitations, we propose a two-stage modified-iKM approach, which provides an improved, accurate, user-friendly, and stable workflow to reconstruct IPD. In this approach, we not only relax the restricted requirement for data input, but also improve the robustness and stability of the original iKM method. More importantly, we develop an all-in-one software platform that allows clinicians and medical researchers to go from a single K-M curve image directly to reconstructed IPD, without using additional software to aid data extraction or without manual treatment on the data before reconstruction. The software includes an *R* package and a web-based Shiny application. Specifically, users can use either the *R* package or the Shiny application to (1) extract data coordinates from published K-M curves; (2) preprocess extracted data points; (3) run the modified-iKM algorithm to estimate the number of patients at risk, the number of events, and the number of censored outcomes for each pre-specified interval, and to reconstruct IPD; (4) provide graphs and statistical summary for users to evaluate the accuracy of the reconstruction process; and (5) conduct survival analysis based on reconstructed IPD. The *R* package is beneficial for users with some programming skills, as they can perform the reconstruction of IPD within *R* or expand their future research on the package. The advantages of the Shiny application are that it has a point-and-click interface, does not require installing any software, and can be used on any machine with an internet browser. This feature is appealing for clinicians and medical researchers who are not necessarily familiar with statistical programming. Extensive simulations and real-world data applications demonstrate that the proposed method has high accuracy and great reliability in the estimations of the parameters of interest, e.g., median survival, hazard ratio, and survival probability.

Our work is of great importance because it not only further supplements and strengthens the original iKM algorithm, but it also provides a user-friendly, all-in-one software available in different platforms to accommodate the needs of researchers with or without statistical programming familiarity. The availability of the two different software platforms can further enhance quality extraction of IPD for meta-analysis and other secondary analyses using time-to-event data.

## Implementation

**Fig. 1:**
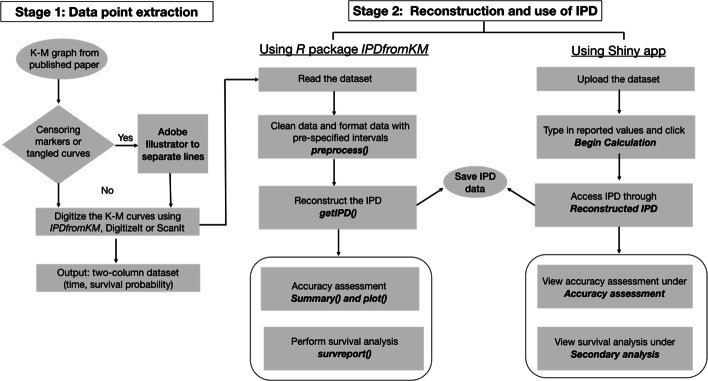
The flowchart of IPD reconstruction from published K-M curves

The modified-iKM algorithm for IPD reconstruction is a two-stage process, as shown in Fig. 1. Stage 1 aims to extract quality data coordinates (*time*, *survival probability*) from K-M curves. In Stage 2, the data coordinates are preprocessed and IPD is reconstructed using the proposed iterative algorithm.

### Coordinates extraction in Stage 1

Data coordinates can be extracted from K-M curves using the *R* package and Shiny application we develop (details in the “Results” section). There are also a number of other software options available on Windows or Mac operating systems to digitize the graphs. The commonly used software are DigitizeIt (http://www.digitizeit.de/), ScanIt (https://www.amsterchem.com/scanit.html), and Plot Digitizer (http://plotdigitizer.sourceforge.net/). Extensive applications of real-world trial examples and simulated K-M curves show that data extracted using the different approaches yield comparable results during IPD reconstruction in Stage 2 (more details in the “Implementation” and “Simulation result” sections). We provide video tutorials on how to extract data coordinates using these tools in our Shiny application.

To ensure accuracy of estimation in Stage 2, it is critical to extract quality data coordinates in Stage 1. We recommend the following when extracting data points:

1. Use Adobe Illustrator to separate the curves before extracting data points, when there are multiple K-M curves in the same figure and the curves are tangled together. A video tutorial on this is included in the “Shiny application” section.
2. Extract as many points as possible.
3. Make sure the data points extracted are as evenly distributed as possible on the K-M curves.
4. Click on both the top and bottom points of the vertical segment at points where survival probability drops.

### IPD reconstruction in Stage 2

The IPD reconstruction is carried out using the modified-iKM algorithm, which is based on the K-M estimation method [9] and improves upon the iKM algorithm [5]. We provide an overview of the algorithm in this section and delineate it in detail in Appendix Modified-iKM algorithm in details. Let *T*_*k*_ and *S*_*k*_ denote the time and survival probability, respectively, at time *k*. The data points extracted in Stage 1 are typically a *N*×2 table, with each row being (*T*_*k*_,*S*_*k*_), for *k*=1,2,...,*N*, where *N* is the number of data points extracted. The IPD reconstruction consists of two main steps as follows:

1. Process the raw data.
  a Sort the data by *T*_*k*_.
  b Make monotonicity adjustment by first using Tukey’s fence [10] to detect and remove unreasonable inputs, and then ensure that survival probability decreases over time (known as “force monotonicity” hereafter).
  c Perform step control.
2. Reconstruct IPD using an iterative algorithm adapted from the iKM method.
  a Estimate K-M parameters at each coordinate (*k*) including the number of patients at risk ($\hat {n}_{k}$), number of patients censored ($\widehat {cens}_{k}$), and number of events($\hat {d}_{k}), k=1,\cdots, N$. In this step, we modified the boundary setup for the number of censored observations to prevent abysmal estimations in some scenarios (more details in Appendices Modified-iKM algorithm in details-Robustness and stability of the modified-iKM algorithm).
  b Construct IPD using the parameters estimated from Step (2a).

Tukey’s fence in Step (1b) is a nonparametric method for outlier detection. It is calculated by creating a “fence” boundary using *TF*=[*Q*_1_−*k*(*Q*_3_−*Q*_1_),*Q*_3_+*k*(*Q*_3_−*Q*_1_)], where *Q*_1_ and *Q*_3_ are the first and third quartiles of the data points, and *k* is a constant that takes common values of 1.5 or 3. We used *k*=3 in our algorithm in order to not accidentally delete useful data points. Any point outside of *TF* is considered an outlier. The illustrative example in Appendix: Robustness and stability of the modified-iKM algorithm shows that the use of Tukey’s fence in Step (1b) improves the robustness of the algorithm in the presence of outliers (Fig. 5 in Appendix). Step (1c) safeguards the algorithm from a potential under-estimation problem (details in Appendix: Modified-iKM algorithm in details). Step (2a) enhances the stability of the iterative algorithm. In Appendix: Robustness and stability of the modified-iKM algorithm, we demonstrate the robustness and stability of the modified-iKM algorithm using two data applications.

To assess the accuracy of the modified-iKM algorithm, we employ several metrics and make them easily accessible through our developed software. First, we provide graphs to visualize the reconstructed results: (1) estimated survival probability at each read-in time point using the reconstructed IPD compared with the corresponding read-in survival probabilities, and (2) estimated number of patients at risk (when given) compared with reported values. Second, we provide several summary statistics to aid the accuracy assessment. One is the root mean square error (RMSE), which measures the difference in survival probabilities calculated using reconstructed data and original data. Additionally, the mean absolute error and the max absolute error are provided to assess the precision of the estimation. Upon careful assessment of the empirical distributions of the RMSE, max absolute error, and mean absolute error for all of the real trial examples and simulated curves considered later in this study, we recommend the following thresholds: RMSE ≤0.05, mean absolute error ≤0.02, and max absolute error ≤0.05 to indicate that the extracted data points are sufficiently well-captured for subsequent analyses. Please note that these recommendations are based on a limited number of examples. In practice, users can use these thresholds as a rule of thumb along with their own judgement to determine if they need to re-extract the data points. Third, we use the Kolmogorov-Smirnov test to compare the distributions of the read-in and the estimated survival curves. A large *p*-value is desired, as it indicates that there is a lack of statistical evidence to show the discrepancy between the read-in and the estimated survival curves.

## Results

### *R* package

To facilitate the use of the modified-iKM method, we developed an *R* package called “*IPDfromKM*” with *R* version 3.6.0. The package is available via the Comprehensive *R* Archive Network (CRAN) at https://CRAN.R-project.org/package=IPDfromKM. The package contains several functions; the descriptions and objects returned for the functions are presented in Table 1. We provide an example for each of the functions below.

**Table 1:** Overview of the user visible functions in *IPDfromKM*

Function	Description	Object returned
getpoints	Extract raw data coordinates from published K-M curves.	A data frame containing the x- and y- coordinates of the K-M curve of interest.
preprocess	Preprocess the read-in data coordinates.	A list including cleaned data ready for reconstruction and a “riskmat” table displaying the index of read-in points within each time interval.
getIPD	Estimate the IPD.	A list including the reconstructed IPD.
survreport	Perform survival analysis on reconstructed IPD.	K-M curve, cumulative hazard, times for targeted survival probabilities.
plot	Plot the object returned by getIPD().	K-M curves and number at risk for both reconstructed IPD and read-in data.
summary	Summarize objects returned by getIPD().	Descriptive results for accuracy assessment and survival analysis on reconstructed IPD.

Please consult the documentation (e.g., help(“preprocess”)) for function arguments and detailed return types

#### Extract data coordinates

The *getpoints()* function is used to extract data coordinates from published K-M curves. The function has the following arguments:

- f: the K-M curves in a bitmap image (e.g.,.png,.jpeg,.bmp,.tiff).
- x1: the actual label of the left-most points on x-axis.
- x2: the actual label of the right-most points on x-axis.
- y1: the actual label of the lowest point on y-axis.
- y2: the actual label of the highest point on y-axis.

For the image, the use of a.png file is highly recommend, since it can shorten the processing time in *R*. In addition to the image itself, two x-coordinates (x1 and x2) and two y-coordinates (y1 and y2) are needed to decide the location and scale of the coordinates system. Below is an example to read in an image for data extraction, where 60 is the rightmost label on the x-axis.


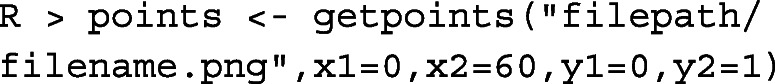


After the file is read into *R*, instructions will be provided in the *R* console to guide the extraction of data points. Specifically, users will be guided to click on the leftmost and rightmost points on the x-axis, and click on the lowest and the highest points on the y-axis. Then they can collect the data coordinates by mouse-clicking on the curve. To get a desirable estimation, we suggest that users follow recommendations 2-4 in the “Coordinates extraction in Stage 1” section. The data points extracted will be returned as a two-column data set (e.g., *points* in the example), and this data set can be used as the input of the *preprocess()* function described in the “Process data coordinates” section.

In the following text, we take the build-in data set *Radiationdata* to demonstrate the use of the package to preprocess raw data, reconstruct IPD, and conduct secondary analysis on the reconstructed IPD. The data set was extracted from published K-M curves from a two-arm randomized controlled trial [11] using ScanIt. This study randomized 424 head and neck cancer patients to two treatment groups: 213 in the radiotherapy group (referred to as “radio”) and 211 in the radiotherapy plus cetuximab group (referred to as “radio_plus”). The primary outcome was the duration of locoregional control. There were 145 pairs of coordinates extracted for the radio treatment, and 136 pairs for the radio_plus treatment. Coordinates for each treatment were saved as a two-column table in *Radiationdata*: the first column is for survival times, and the second column is for survival probabilities reported in percentages. *Radiationdata* also includes risk times (in months): *trisk*=(0,10,20,30,40,50,60,70) and the number of patients at risk for each risk time point (*nrisk*).

#### Process data coordinates

To prepare data to reconstruct IPD, we first preprocess the raw data points in an appropriate format. This can be done using the *preprocess()* function. This function contains the following arguments:


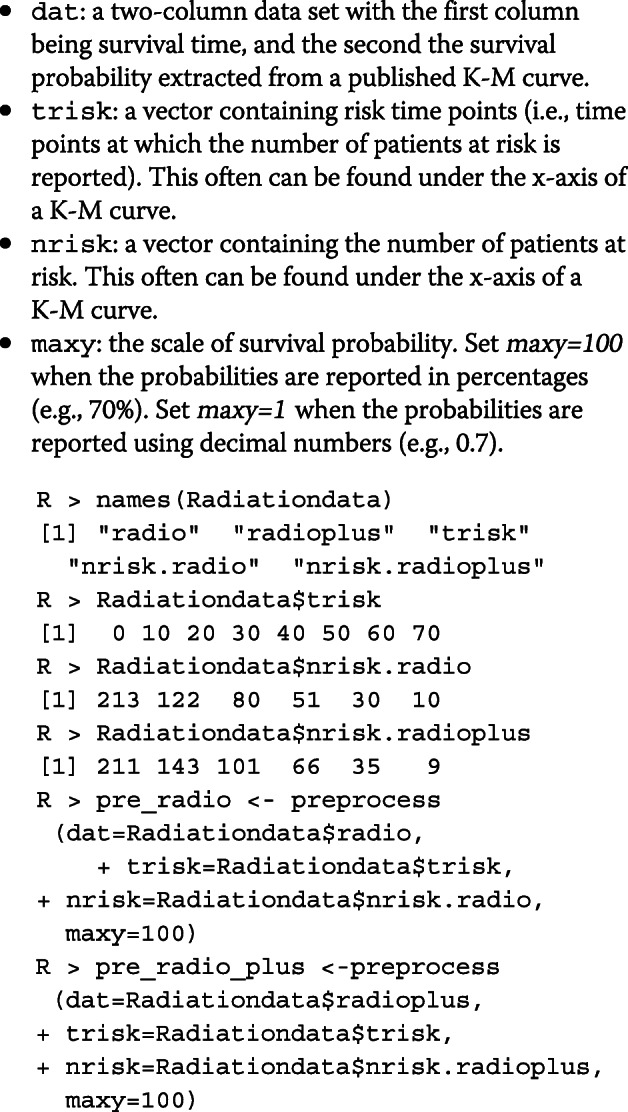


The output of the *preprocess()* function is a class object that can be used directly in the *getIPD()* function to construct IPD.

#### Reconstruct IPD

After the raw data is processed using the *preprocess()* function, we can use the *getIPD()* function to reconstruct the IPD. The *getIPD()* function has the following arguments:

- prep: the class object returned from the *preprocess()* function.
- armID: an arbitrary label used as the group indicator for the reconstructed IPD. Typically 0 for the control group and 1 for the treatment group.
- tot.events: total number of events. Only available for some published curves, and the default value is NULL.

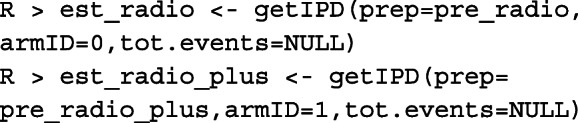


#### Accuracy assessment

To view the accuracy assessment results, simply call the *summary()* function. Because of page limits, we show only one example below.


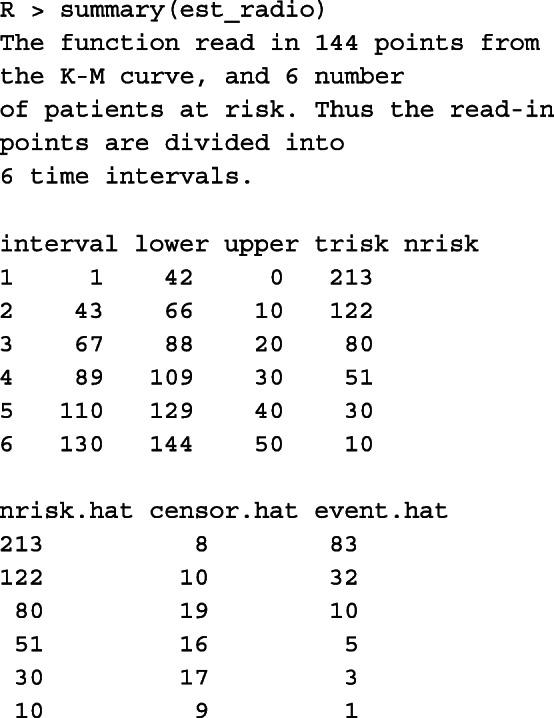


We see that our algorithm can accurately estimate the numbers at risk and provide estimates on the number of events at each risk time. The small values for RMSE (<0.05), mean absolute error (<0.02), and max absolute error (<0.05), along with the large *p*-value of the Kolmogorov-Smirnov test shows that the reconstructed IPD is accurate. Additionally, we can use the *plot()* function to graph the survival curves from the reconstructed IPD, and compare them with those generated using original data points. The function takes the object returned by *getIPD()* directly.


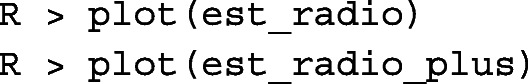


The output using the *plot()* for the radio group is provided in Fig. 6 (Appendix: Figures returned for accuracy assessment or secondary analysis), which shows three graphs: (1) K-M curves based on reconstructed IPD and read-in data points, respectively; (2) number of patients at risk using the reconstructed IPD versus reported; and (3) difference in survival probability at different time points for the reconstructed IPD and the read-in data. When the interval information is not available while reconstructing the IPD, the second graph will not be shown.

#### Secondary analysis

If survival analysis is of interest, we can run the *survreport()* function, which includes the following arguments:

- ipd1: a three-column (i.e., time, status, treatment indicator) table of IPD for treatment 1.
- ipd2: a three-column (i.e., time, status, treatment indicator) table of IPD for treatment 2.
- arms: number of treatment arms (value of either 1 or 2).
- interval: the time intervals for which the landmark survival probabilities are of interest. The default is at every 6 months.
- s: the survival probabilities for which the corresponding survival times are of interest, e.g, s=0.5 means that the median survival time is desired.

Researchers working with clinical data are often interested in the survival times at which survival probabilities are specified (e.g., median survival time at which 50% of patients survive). The example below shows the survival times for the pre-specified survival probability *s*=(0.50,0.75,0.95). This function also returns K-M curves and cumulative risk in a figure for both groups (Fig. 7 in Appendix: Figures returned for accuracy assessment or secondary analysis).


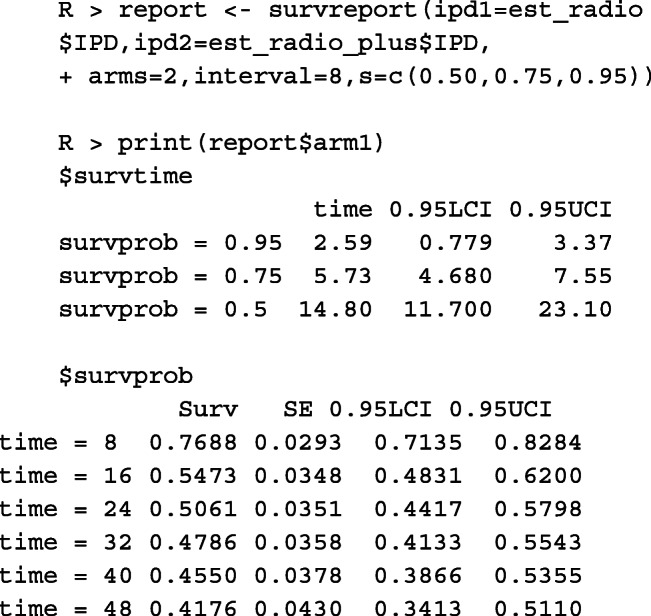


### Shiny application

To facilitate the use of the modified-iKM algorithm to reconstruct IPD for a broader audience, who is not necessarily familiar with statistical programming, we develop a user-friendly Shiny application, which is freely available at https://www.trialdesign.org/one-page-shell.html#IPDfromKM. Compared with the *R* package, the Shiny application does not require any installation, is not limited by the hardware of users’ computers, and comes with a point-and-click feature such that no statistical programming skill is needed to use it [12]. In addition, we regularly maintain and update the application on the server, thus users do not have to worry about software updates. Whenever users have any questions, they can contact the authors directly through the Shiny application host website. Appendix: Long-term commitment of the shiny application shows how users can directly contact the authors.

The application has a straightforward interface with three main panels: **Data Extraction** (used to extract data points), **Reconstruct Individual Patient Data (IPD)** (used to reconstruct IPD), and **User Guide** (providing extensive tutorials). Overall, the app has the capability to complete the following tasks:

1. Extract data points from published K-M curves.
2. Process extracted data points.
3. Reconstruct IPD using the modified-iKM algorithm.
4. Assess the accuracy of the reconstruction.
5. Perform survival analysis using the reconstructed IPD.
6. Generate a concise report for the IPD reconstruction.
7. Provide an extensive user guide for understanding the method and using the Shiny application.

Typical input required to use the app includes the following, where input 1 is for data extraction and inputs 2-6 are for IPD reconstruction.

1. An image file (.png or.jpeg) containing the K-M curve of interest.
2. A.csv or.txt file containing the coordinates extracted from published K-M curves (for IPD reconstruction). File templates are available in the Shiny application.
3. Risk time (*trisk*).
4. Number of patients at risk (*nrisk*).
5. Total number of patients reported (optional when information for *nrisk* is provided).
6. Total number of events reported (optional, but having it will improve accuracy).

We provide video tutorials on how to extract coordinates using the Shiny application and other software, and how to reconstruct IPD from extracted data coordinates. The tutorial are accessible under the **User Guide** panel of the Shiny application. In the following text, we show an example of using the Shiny application to reconstruct IPD for two treatment groups simultaneously.

**Fig. 2:**
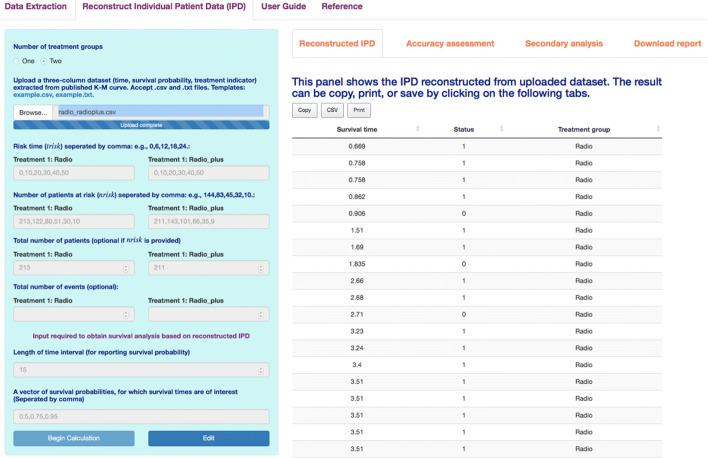
IPD reconstruction of two treatments using the Shiny application

We illustrate the use of the application using data for the radio and radio_plus treatment groups introduced in the *R* package examples. The data set saved in the *radio_radioplus.csv* file has been provided in the app as a template. As shown in Fig. 2, to input the data, we select “Two” under *Number of treatment groups* and upload the data file. We then type in available information including risk times, number of patients at risk, and the total number of patients for each group. When finishing data input, we simply click the *Begin Calculation* button and the results are shown on the right side of the app. There are four tabs for displaying the results.

As shown in Fig. 2, the first tab **Reconstructed IPD** shows the individual patient data reconstructed from the data provided. The **Accuracy assessment** tab (Fig. 10 in Appendix) shows two plots and a table. The first plot displays the comparison of K-M curves using the IPD and original data set for each treatment group, followed by a plot of the difference between survival probabilities. The table shows the summary statistics such as RMSE and Kolmogorov-Smirnov test statistics and *p*-values to help assess the accuracy of the IPD reconstruction. Under the **Survival analysis** tab (Fig. 11 in Appendix), K-M curves and cumulative hazard functions are displayed for both treatments. For each treatment, the landmark survival probabilities, corresponding standard error, and 95% confidence interval (CI) are reported. Below the landmark probability tables, the application also provides the critical survival times given pre-specified survival probabilities. For example, we see that the median survival time is 14.9 months for treatment 1 (radio group) and 24.5 months for treatment 2 (radio_plus group). A concise report is available under the **Download report** tab (Fig. 12 in Appendix).

### Implementation example

We now illustrate the use of the modified-iKM method for reconstructing IPD from K-M curves published for a two-arm randomized controlled trial in non-small cell lung cancer (NSCLC) patients [13]. This trial is known as the POLAR trial, in which a total of 287 patients with previously treated, advanced or metastatic NSCLC were randomized to receive either atezolizumab or docetaxel. The aim of this trial was to assess the efficacy of the two drugs for patients with NSCLC, analyzed by PD-L1 expression levels on tumor cells and tumor-infiltrating immune cells. Here, the baseline PD-L1 expression was scored by immunohistochemistry in tumor cells (as the percentage of PD-L1 expressing tumor cells TC3 (≥50%), TC2 (≥5% and <50%), TC1 (≥1% and < 5%), and TC0 (<1%)) and tumor-infiltrating immune cells (as the percentage of tumor area: IC3 (≥10%), IC2 (≥5% and <10%), IC1 (≥1% and <5%), and IC0 (<1%)). Overall survival was estimated for all patients in five groups: TC3 or IC3 patients, TC2/3 or IC2/3 patients, TC1/2/3 or IC1/2/3 patients, and TC0 or IC0, and intention to treat for both atezolizumab and docetaxel treatments. The five pairs of K-M curves in the POLAR trial are shown in Fig. 8 (Appendix: Original K-M curves in the POLAR trial).

To apply our method and assess its accuracy, we extract raw data points from the K-M curves, reconstruct IPD from the points extracted, use the reconstructed IPD to calculate the median overall survival (OS) and hazard ratio, and compare them with the published results. As shown in Table 2, the point estimates for the median OS and HR are close to reported values based on raw data.

**Table 2:** Estimates of median overall survival (OS) and hazard ratio using the modified-iKM algorithm based on data extracted using different software (*R*: *R* package *IPDfromKM*, *D*: DigitizeIt, *S*: ScanIt), in comparison to published results (Report) in the POLAR trial

			Median OS						Hazard Ratio			
Group	Arm	*n*	Report	R	D	S			Report	R	D	S
1	Atezolizumab	24	15.5	15.5	15.5	15.5			0.49	0.48	0.46	0.45
	Docetaxel	23	11.1	11.1	11.1	11.1						
2	Atezolizumab	50	15.1	15.3	15.3	15.3			0.54	0.54	0.56	0.53
	Docetaxel	55	7.4	7.6	7.4	8.1						
3	Atezolizumab	93	15.5	15.3	15.5	15.7			0.59	0.59	0.58	0.59
	Docetaxel	102	9.2	9.3	9.2	9.6						
4	Atezolizumab	51	9.7	9.7	9.7	9.5			1.04	1.06	0.99	1.03
	Docetaxel	41	9.7	9.7	9.7	9.8						
5	Atezolizumab	144	12.6	13.3	12.4	12.3			0.73	0.72	0.70	0.72
	Docetaxel	143	9.7	9.7	9.7	9.8						

Group 1: TC3 or IC3; 2: TC2/3 or IC2/3; 3: TC1/2/3 or IC1/2/3; 4: TC0 or IC0; 5: all patients. The value of *n* refers to sample size

Considering that the reconstructed IPD data may not be independent and that data extraction may bring up extra variation in the reconstruction process, it is of interest to see how the variability of test statistics based on the reconstructed IPD impacts the hypothesis testing of interest. We evaluated this by conducting hypothesis tests using the reconstructed IPD from the five pairs of K-M curves in the POLAR trial and six additional pairs of simulated K-M curves (generated in “Simulation result” section). As shown in Table 3 (Appendix: Discussion on variability of test statistics), our method, with reconstructed IPD, reaches the same conclusion as that using the true IPD, indicating that results based on reconstructed IPD are reliable. Moreover, we further investigated the variability of statistics of interest (e.g., hazard ratio) between using true IPD and using reconstructed IPD, through bootstrap confidence intervals. As shown in Table 4 (Appendix: Discussion on variability of test statistics), the variability of statistics based on reconstructed IPD is comparable to that based on true IPD. The examinations on the variability of test statistics in our study are limited. Future studies are warranted to further investigate this aspect. In terms of software used, we see that the data points extracted using our function from the *IPDfromKM* package have competitive performance to those extracted using DigitizeIt and ScanIt. In practice, users can use the software of their choice as long as they follow the instructions to carefully extract data points. Nevertheless, the all-in-one feature of our software greatly streamlines the data extraction, IPD reconstruction, and subsequent data analysis, making it a superb choice for users who wish to do everything within one platform.

### Simulation result

To further assess the accuracy of the estimations using the modified-iKM algorithm, we conducted a simulation study with six trials that each had both control and treatment groups. The sample size for each of the 12 groups was 200. We generated the underlying survival time (*T*_*i*_,*i*=1,⋯,200) using the Weibull distribution with the survival function *S*(*t*)=*exp*(−*λt*^*γ*^), where *λ* and *γ* are the scale and shape parameters, respectively [14]. When *γ*>1, the survival curve has an increasing hazard; when *γ*=1, the survival curve has a constant hazard; and when *γ*<1, the survival curve has a decreasing hazard.

In the simulation, the scale parameter was specified such that the mean survival *E*(*X*)=*λΓ*(1+1/*γ*) was 12 months for treatment groups, and 6 months for control groups. We assume 36 months of recruiting time, and a maximum of 24 months of follow-up. The censoring time was generated by the minimum value of the time generated from exponential distribution with parameter *λ*^∗^ and the maximum follow-up time. The value of *λ*^∗^ was set such that the censor rate was either 30% or 60%. K-M curves were then generated by the *survfit()* function from the *survivalR* package. The number of patients at risk was reported every 3 months (20 intervals), or every 10 months (6 intervals), or not reported at all (no risk information). The simulated trials represented diverse situations that mimicked K-M survival curves obtained from real trials.

**Fig. 3:**
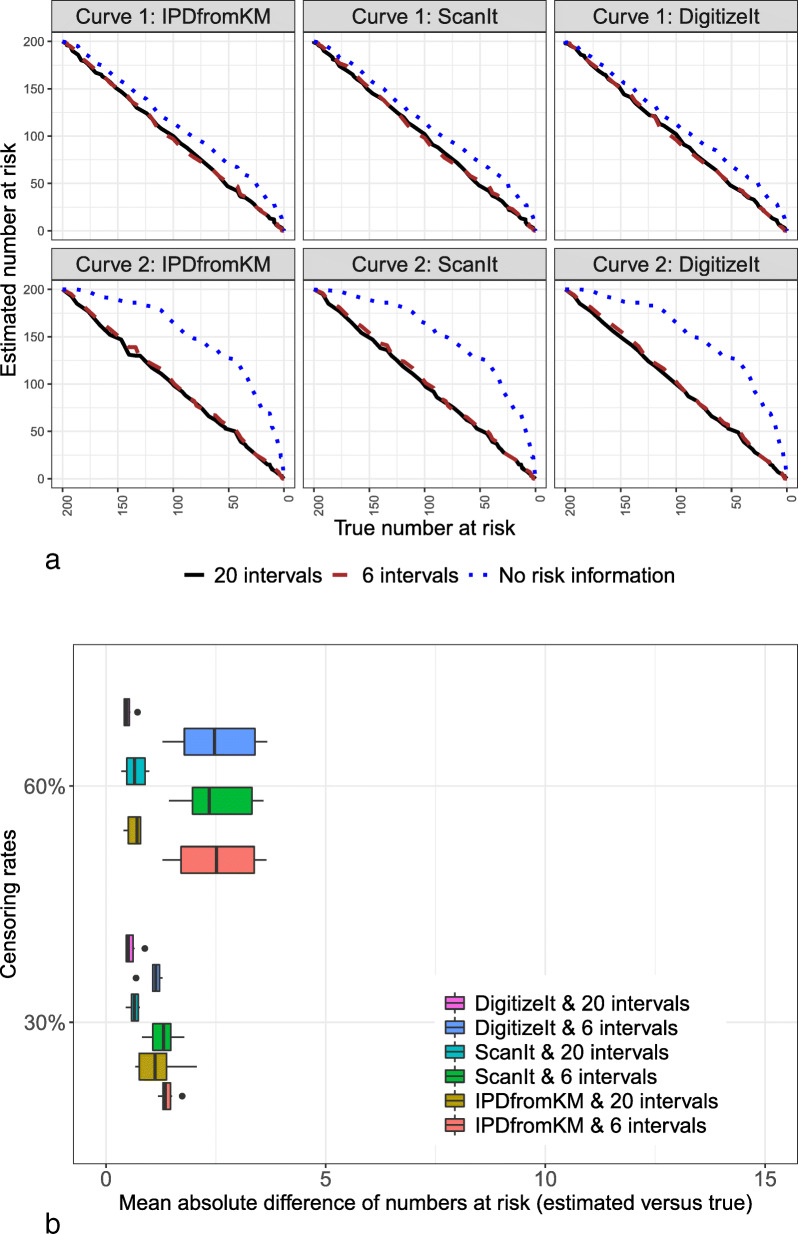
Accuracy assessment in terms of the number of patients at risk

We used the *IPDfromKM* package, DigitizeIt, and ScanIt software to extract the coordinates of the curves, then we used the *preprocess()* and *getIPD()* functions from the *IPDfromKM* package to process the raw data and obtain IPD. We compared the estimated IPD with the true IPD generated. We first examined the accuracy of the three software packages in the coordinates extraction by comparing the estimated number at risk to the true number at risk. Figure 3 shows the results for two simulated curves. As demonstrated, modified-iKM accurately estimated the number of patients at risk, regardless of software used to extract data points, as long as a study reported the number of patients at risk, under both low and high censoring rates. Without reported numbers at risk, estimation cannot be accurate, as previously noted in the literature [5]. Thus, reporting this information in published studies is highly recommended.

Figure 3b shows the mean absolute error of the estimation for the number of patients at risk when the reported number at risk was available (i.e., the case with 6 or 20 time intervals in the trial), where the error was determined by (estimated value − true value). The data extracted yielded comparable results for the different software. The results were more accurate with more time intervals provided. For instance, when there was information for 20 intervals, the mean absolute error was less than 2 regardless of censoring rate. When the censoring rate was large (e.g., 60%), the estimation using information from six intervals had greater error.

**Fig. 4:**
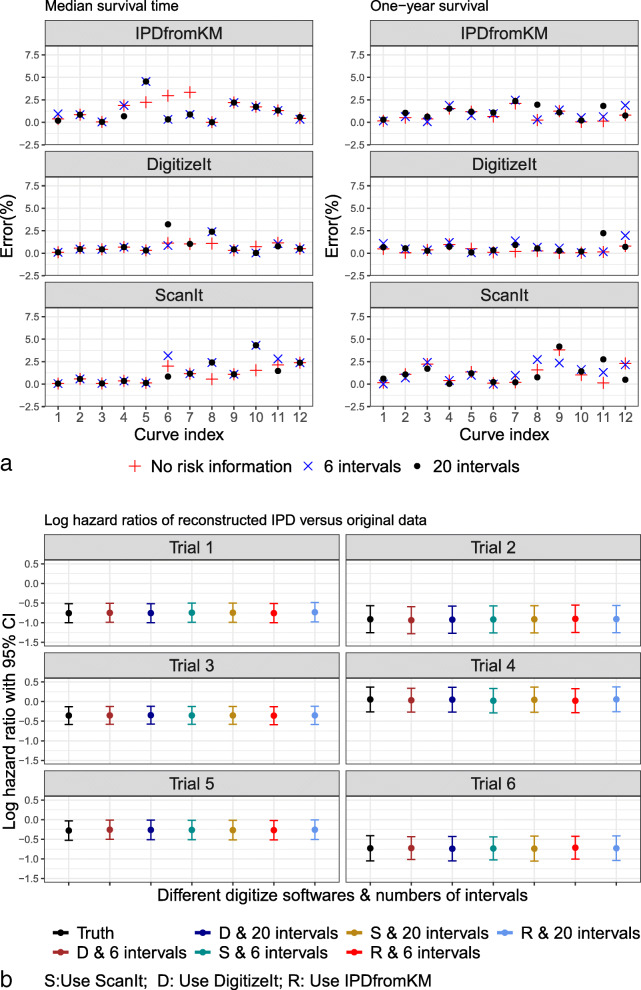
Survival analysis on the reconstructed IPD and the true data

Next, we used the reconstructed IPD to conduct standard survival analysis to evaluate the performance of our method. Figure 4a shows the estimated median survival times and the one-year survival probability. The difference between the estimated and true median survival times was within 5% in most cases. Figure 4b shows the comparison between the true hazard ratio and estimated hazard ratios on the log scale. The hazard ratios and corresponding standard deviations were almost identical in most cases, regardless of which software was used to extract the data coordinates.

## Conclusion

In this work, we introduce the *R* package *IPDfromKM*, and its accompanying Shiny application, to reconstruct IPD from published K-M curves based on the proposed modified-iKM algorithm. There are several improvements in the modified-iKM algorithm in comparison to the original iKM method. First, we provide a function for users to extract data points from K-M curves directly. This enables the all-in-one feature of our software, which streamlines the process of IPD reconstruction without requiring extra software to obtain the raw data coordinates. Our real-world examples and simulated examples show that data points extracted using the *IPDfromKM* package yield competitive results to those extracted using DigitizeIt and ScanIt as long as points extracted are carefully extracted.

Second, our procedure to preprocess raw data coordinates is flexible and accurate. The original iKM method does not have a function for preprocessing raw data coordinates, and users need to manually check the monotonicity assumption and sort the extracted data points. Stagopan et al.[6] published a function that can automatically preprocess the raw data coordinates. However, their function tends to be unstable. For example, the function simply deletes points that have a larger survival probability than the points right before them, which introduces additional errors into the extracted data points. The function also trims data points at the tail of a K-M curve. This can be detrimental, as the K-M curve often has a long horizontal tail. If data points were trimmed at the tail, it would be less likely to reliably estimate the number of censored observations occurring at the end of the trial. The *preprocess()* function from the *ReconstructKM* package published on GitHub [8] can preprocess the data coordinates but it also requires manual check for monotonicity and the curve endings. To overcome these limitations, we proposed a flexible and accurate algorithm to preprocess the raw data coordinates extracted from K-M curves and the algorithm is easily implemented by using the *preprocess()* function from the *IPDfromKM* package or by uploading the data to the Shiny application. Moreover, the use of Tukey’s fence improves the robustness of reconstruction in Stage 2 in the presence of outliers.

Third, in the estimation stage, we refine the boundary setup in the iterative procedure to prevent abysmal estimations, which greatly improves the stability of the iKM method.

Finally, we demonstrate through simulation that the variability of statistics of interest is comparable to that based on the true IPD and that the conclusion based on reconstructed IPD is the same as that based on the true IPD in hypothesis testing. These findings show that the IPD reconstructed using our method is reliable. In the *R* package and the Shiny application, we provide several approaches to evaluate the accuracy of the algorithm with an easy-to-use function, with which the quality of the reconstructed IPD is easily assessed.

Despite the strengths of the modified-iKM algorithm and its accompanying software, there are a couple of challenges or limitations worth mentioning. First, when there are tangled lines with a lot of censoring markers, even with the help of software such as Adobe Illustrator, it is still challenging to separate out each K-M curve for digitizing. Second, while the modified-iKM algorithm can accurately estimate the survival probabilities, the number of events, and the number of patients at risk, the algorithm is based on a uniform censoring assumption, which may be violated in certain trials. Future work can expand on this and consider extension to a non-uniform censoring mechanism.

## Availability and requirements

Project name: IPDfromKM R package and Shiny web application.

Project home page: https://CRAN.R-project.org/package=IPDfromKM, and https://www.trialdesign.org/one-page-shell.html#IPDfromKM.

Operating system(s): Platform independent.

Programming language: R.

Other requirements: none.

License: GPL-2.

Any restrictions to use by non-academics: none

## Appendix

### Modified-iKM algorithm in details

The IPD reconstruction is carried out using the modified-iKM algorithm, which is based on the K-M estimation method [9] and improves upon the iKM algorithm [5]. Details regarding the iKM estimation method and the modified-iKM algorithm are provided below.

#### The KM estimator

The KM estimator, first proposed by Kaplan and Meier [9] is a non-parametric estimator of the survival function. It is determined by the product over the failure times of the conditional probabilities of surviving to the next failure time. Specifically, suppose there are *Q* distinct failure times. Let *t*_*q*_ denote a time where at least an event (e.g., a patient dies) is observed, *n*_*q*_ is the number of subjects at risk at time *t*_*q*_, and *d*_*q*_ is the number of subjects who experience the event at that time, *q*=1,⋯,*Q*. The KM estimator ($S^{KM}_{t_{q}}$) is formally defined as

1 $$ S^{KM}_{t_{q}}=\prod_{j=1}^{q}\frac{1-d_{j}}{n_{j}}=S^{KM}_{t_{q-1}}*\frac{1-d_{q}}{n_{q}}, q=1, \cdots, Q.   $$

The number of patients at risk (*n*_*q*+1_) can be determined by the number of patients at risk at time *t*_*q*_, minus the corresponding number of patient experienced events and number of patients censored.

2 $$ n_{q+1}=n_{q}-d_{q}-c_{q}.   $$

#### The modified-iKM algorithm

Before initializing the iterative algorithm, we first sort extracted coordinates by time and make monotonicity adjustments on survival probabilities. In addition, to improve estimation accuracy, we propose a step control procedure while calculating the number of events at the coordinate *k* (denoted as $\hat {d}_{k}$). Usually, there will be multiple points at the same time, and thus multiple survival probabilities are available at this time. In a K-M curve, this is reflected by a drop in the survival probability at a time point. We refer to such a time point as a jumping step. Suppose there are *s* consecutive points read out at the jumping step from the target K-M curve, and denote them as *j*,*j*+1,...*j*+*s*−1. The original iKM method requires the use of all the values for $\hat {d}_{j}, \hat {d}_{j+1},...,\hat {d}_{j+s-1}$ and adds them together to get the estimated total number of events that happened at the jumping step. However, this could end up in an under-estimated number at risk due to rounding. That is, $\hat {d}_{j}, \hat {d}_{j+1},...,\hat {d}_{j+s-1}$ can be too small to round off to zero. When too many jumping points exist, the number at risk is under-estimated. As a simple illustrative example, suppose *s*=10, if all the values for $\hat {d}_{j}, \hat {d}_{j+1},...,\hat {d}_{j+s-1}$ are within [0.1,0.5). After rounding these values separately and adding them together, the estimated number at risk is zero, but in reality, it lies between 1 and 5. We note that this rarely happens in practice based on extensive simulations and real trial examples. Nevertheless, it is great to safe guard against the potential under-estimation problem. For this reason, we recommend controlling the steps to ensure that there are only two points remaining in each vertical segment on the K-M curve: one at the beginning and the other one at the end of the segment. The corresponding survival rates at these two points are simply $\hat {S}^{KM}_{last(k)}$ and *S*_*k*_. By doing this, the number of events at this step needs to be calculated and rounded only one time, and it would not end up with the potential under-estimated problem.

**Table 3:** Hypothesis testing using log-rank test with true IPD versus with reconstructed IPD based on data points extracted using different software

	Results based			Results based on reconstructed IPD							
	on true IPD			IPDfromKM			DigitizeIt			ScanIt	
	HR(SE)	*p*-value		HR(SE)	*p*-value		HR(SE)	*p*-value		HR(SE)	*p*-value
Simulated curves:											
1	0.47(0.12)	<0.001		0.48(0.13)	<0.001		0.47(0.12)	<0.001		0.47(0.12)	<0.001
2	0.40(0.18)	<0.001		0.40(0.18)	<0.001		0.40(0.18)	<0.001		0.40(0.18)	<0.001
3	0.70(0.12)	0.002		0.70(0.12)	0.003		0.71(0.12)	0.003		0.70(0.12)	0.002
4	1.06(0.16)	0.735		1.06(0.16)	0.727		1.05(0.16)	0.761		1.05(0.16)	0.780
5	0.76(0.13)	0.028		0.77(0.13)	0.044		0.77(0.13)	0.039		0.77(0.13)	0.037
6	0.48(0.16)	<0.001		0.48(0.16)	<0.001		0.48(0.16)	<0.001		0.48(0.16)	<0.001
Real trial example:											
1	0.49(0.22)	0.068		0.48(0.41)	0.064		0.46(0.38)	0.041		0.45(0.41)	0.041
2	0.54(0.14)	0.014		0.54(0.26)	0.017		0.56(0.26)	0.020		0.53(0.26)	0.013
3	0.59(0.11)	0.005		0.58(0.19)	0.005		0.58(0.19)	0.005		0.59(0.19)	0.007
4	1.04(0.29)	0.871		1.06(0.26)	0.830		0.99(0.25)	0.959		1.03(0.26)	0.917
5	0.73(0.12)	0.040		0.72(0.15)	0.031		0.70(0.14)	0.019		0.72(0.15)	0.036

*HR* hazard ratio, *SE* standard error

To start the iterative estimation process, we first divide the preprocessed coordinates from the K-M curve into *I* intervals. Denote the number of patients at risk at the beginning of the intervals as (*nrisk*_1_,*nrisk*_2_,⋯,*nrisk*_*I*_) and denote the time at which the number of patients at risk is provided as (*trisk*_1_,*trisk*_2_,⋯,*trisk*_*I*_). For each interval, we denote the index of the first point as *lower*_*i*_ and of the last point as *upper*_*i*_. The iterative estimation process proceeds as follows:

1. Initialize the total number of patients censored at interval *i* ($\widehat {ncensor}_{i}$) using the difference between the reported number at risk at the beginning of interval *i*+1 and the number at risk in this interval if no censoring occurs ($nrisk_{i+1}^{nocensor}$) in interval *i*. The value of $nrisk_{i+1}^{nocensor}$ is given by $nrisk_{i+1}^{nocensor}=nrisk_{i}*S_{lower_{i+1}}/S_{lower_{i}}$ rounded to the nearest integer, where $S_{lower_{i+1}}$/$S_{lower_{i}}$ is the probability of survival at the beginning of interval *i*+1 conditional on being alive at the beginning of interval *i*. Thus we have
  3 $$ \widehat{ncensor}_{i}=nrisk_{i}*S_{lower_{i+1}}/S_{lower_{i}}-nrisk_{i+1}.  $$
2. Determine the number of patients censored between the extracted coordinates *k* and *k*+1 (denoted as $\widehat {ncenso}r_{k}$). Assuming a constant censoring rate, it is straightforward to determine the censoring time by distributing the number of censored patients evenly over the interval *i*: $tcensor_{m}=T_{lower_{i}}+m*(T_{lower_{i+1}}-T_{lower_{i}})/(nc\hat {en}sor_{i}+1), m=1, \cdots, \widehat {ncensor}_{i}$. The value of $\widehat {ncensor}_{k}$ is determined by counting the censoring times that lie between [*T*_*k*_,*T*_*k*+1_]:
  4 $$ \widehat{ncensor}_{k}= \sum_{m=1}^{n\hat{en}sor_{i}}I(tcensor_{m}\in[T_{k}, T_{k+1}]),  $$
  where *I*(*tcensor*_*m*_∈[*T*_*k*_,*T*_*k*+1_]) is an indicator function, which returns 1 if the censoring time *tcensor*_*m*_ lies within the interval [*T*_*k*_,*T*_*k*+1_].
3. Determine the number of patients at risk for the coordinate *k*+1 (i.e., $\hat {n}_{k+1}$) as
  5 $$ \hat{n}_{k+1}=\hat{n}_{k}-\hat{d}_{k}-nc\hat{en}sor_{k}, k=lower_{i}, \cdots, upper_{i}  $$
  according to Eq. (2), where $\hat {d}_{k}$ is determined by $\hat {n}_{k}*\left (1-S_{k}/\hat {S}^{KM}_{last(k)}\right)$. Round to the nearest integer, based on an rearrangement of Eq. (1). Here $\hat {S}^{KM}_{last(k)}$, instead of $\hat {S}^{KM}_{k-1}$, is used because there may not be an event at the extracted coordinate *k*−1. Thus, $\hat {S}^{KM}_{last(k)}$ is the survival probability of the last point before time *T*_*k*_.
4. Check if the estimated number at risk at the start of the next interval *i*+1 (i.e., $\widehat {nrisk}_{i+1}$, which is just $\hat {n}_{upper_{i}+1}$) is equal to the reported value *nrisk*_*i*+1_.
  If $\widehat {nrisk}_{i+1}=nrisk_{i+1}$, move to step 5.
  Otherwise, set $$ {\hat{ncensor}}_i:= {\hat{ncensor}}_i+{\hat{n}}_{\left( uppe{r}_i\right)+1}- nris{k}_{i+1}. $$
5. Repeat the iteration in steps 1-4, as long as one of the following two conditions holds:
  6 $$ {}\left(\hat{n}_{(upper_{i})+1} > nrisk_{i+1}\right) \mathbf{and}\! \left(\widehat{ncensor}_{i} \!<\! nrisk_{i} - nrisk_{i+1} \right).  $$
  or
  7 $$ \left(\hat{n}_{(upper_{i})+1} < nrisk_{i+1}\right) \mathbf{and } \left(\widehat{ncensor}_{i} > 0\right).   $$
  The condition in (6) shows that the number of patients at risk for interval *i* is greater than the reported value, so the iteration will continue to increase the number of censored patients for this interval. On the other hand, condition (7) shows that the number of patients at risk at the end of interval *i* is less than the reported value, thus the iteration will continue to decrease the estimation of the number of censored patients for the interval. Note that, in condition (6), the upper bound on the number of censored patients ($\widehat {ncensor}_{i}$) is added (i.e., $\widehat {ncensor}_{i} < nrisk_{i} - nrisk_{i+1}$) to ensure that the number of censored patients for each interval lies within the range of [0,*nrisk*_*i*_−*nrisk*_*i*+1_]. This proper boundary condition is needed to avoid having an infinite number of iterations, which was not considered in the original iKM method.
6. Check if the iteration reaches the last interval.
  If *i*+1 is not the last interval, i.e., *i*+1≠*I*, repeat steps 1-4.
  Otherwise, adjust the initial guess of the censored patients in the last interval as
    8 $$ \begin{aligned} \widehat{ncensor}_{I} &= min(\text{Average censoring rate}*\text{length of}\\ &\text{the last interval}, \\ & nrisk_{I} - endpts - (tot.events - \sum_{k=1}^{upper_{I-1}} \hat{d}_{k})), \end{aligned}  $$
    where *endpts* is the last reported number of patients at risk. The adjustment is made because the number at risk for the last interval (*nrisk*_*I*_) is typically not available, and thus the equation in (3) can cannot be determined directly.

### Robustness and stability of the modified-iKM algorithm

We state that Step (1a) and (2b) of the modified-iKM algorithm improve the robustness and stability of the original iKM. In this section, we show two data applications to demonstrate these advantages.

**Fig. 5:**
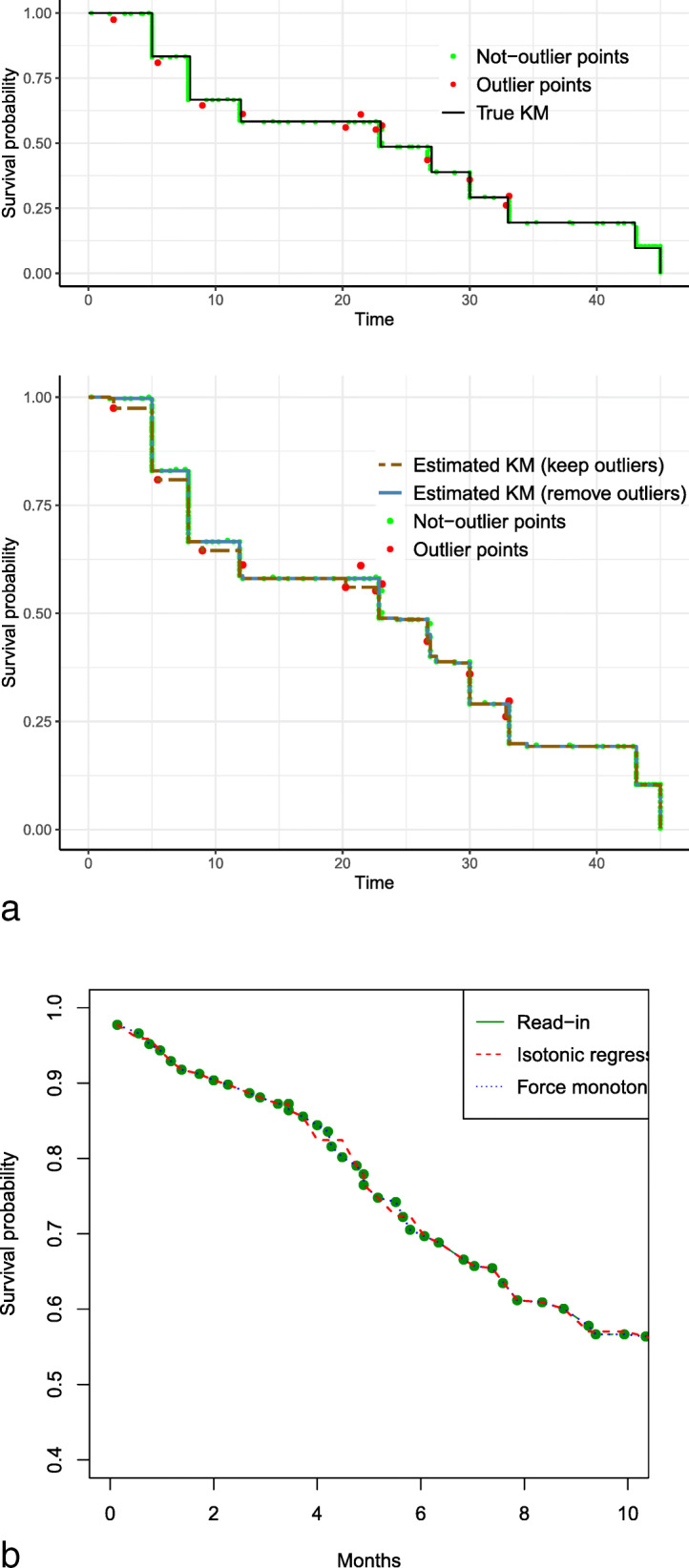
Advantage of Tukey’s fence and “force monotonicity” in Stage 1

**Robustness** We demonstrate the advantage of Tukey’s fence using the data for the acute Myelogenous Leukemia (AML) survival data in the *survival* package. We generated the K-M curve using the data for the control group with the *survminer* package and extracted data points from the K-M curve using the *IPDfromKM* package. The first panel in Fig. 5a shows the data points extracted from the published K-M curve, where the red dots indicate the outliers flagged using Tukey’s fence. The second panel shows the reconstructed K-M curves with and without the outliers. As shown, we can see that by using Tukey’s fence to remove outliers, the algorithm can ensure accurate estimation. This shows that the use of Tukey’s fence increases the robustness of the algorithm such that outliers in the extracted data points will be removed automatically and have a minute impact on the estimation. After removing outliers, we use “force monotonicity” to ensure that survival probability monotonically decrease with time. While isotonic regression is commonly used when monotonicity assumption is violated, we show that force monotonicity is better in most of the curves considered in this study. This may be due to the fact that the former is more likely to smooth out the curve (an example is show in Fig. 5b).

**Fig. 6:**
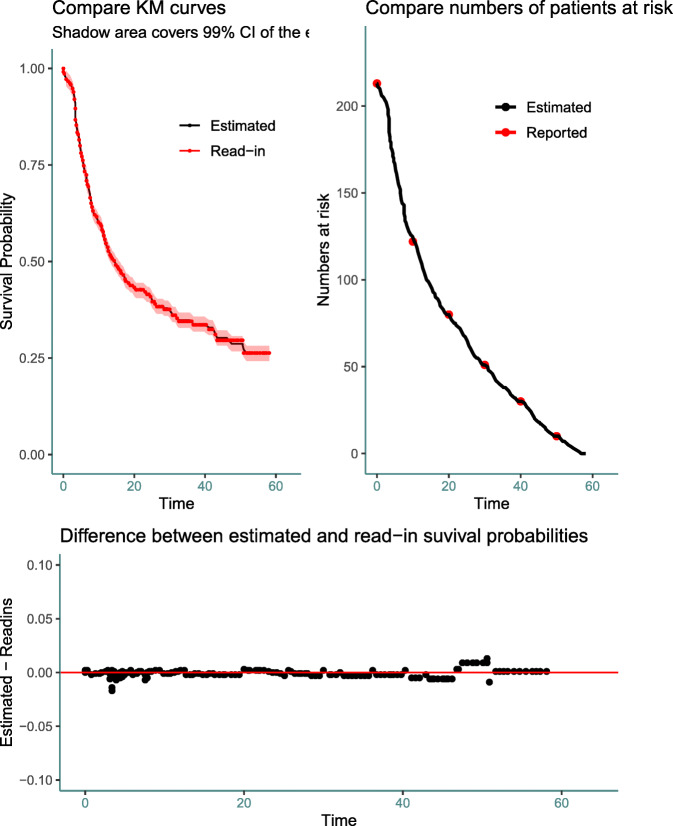
Results of accuracy assessment results using the *plot()* function

**Fig. 7:**
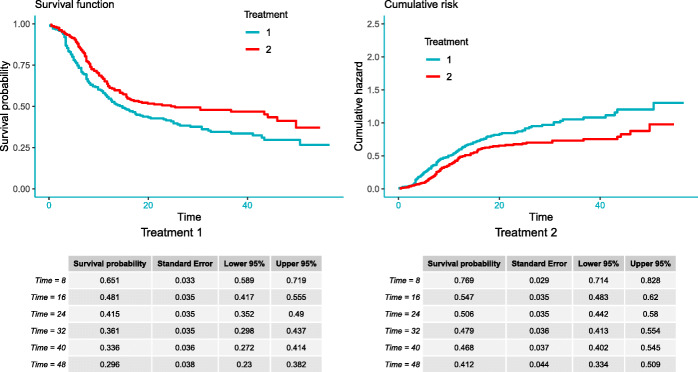
Graphs reported by the *survreport()* function

**Fig. 8:**
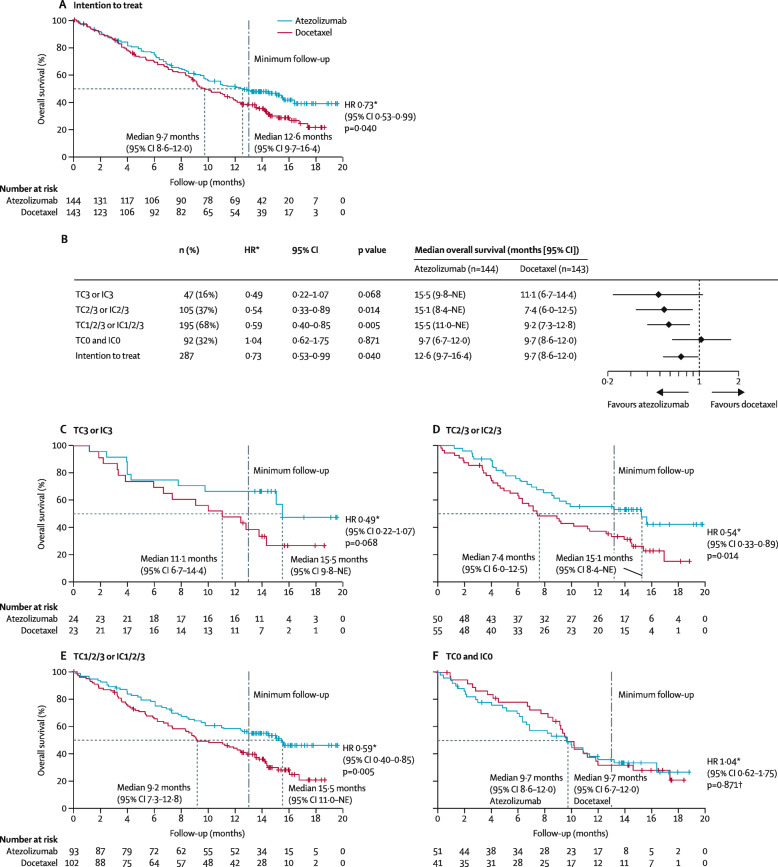
Original K-M curves in the POLAR trial

**Stability** Step (2a) proves to be most useful when there are many reported time intervals. Take the tenth K-M curve in our simulation study for example. We extracted 132 data points from the curve and applied both the modified-iKM and the original iKM methods to estimate the number of patients at risk (nrisk) and number of patients censored at each interval (ncensor). As shown in Table 5, if 6-interval information was used, both modified-iKM and original K-M would produce similar estimations. However, if 11-interval information was used, the modified-iKM can reliably produce $\widehat {ncensor}$ for each interval due to its stability by adding the additional boundary setup (Appendix Modified-iKM algorithm in details). In contrast, the original iKM algorithm behaves abysmally (i.e., negative estimated values). This demonstrates the stability of the modified-iKM.

**Table 4:** Hazard ratio and 95% Bootstrap confidence interval (BCI) for the six simulated trials in Table 3

	Results based	Results based on reconstructed IPD				
Curve	on true IPD		IPDfromKM		DigitizeIt	ScanIt
1	0.469 [0.369, 0.594]		0.487 [0.379, 0.627]		0.470 [0.366, 0.597]	0.474 [0.368, 0.593]
2	0.408 [0.288, 0.554]		0.410 [0.281, 0.570]		0.403 [0.278, 0.566]	0.407 [0.277, 0.546]
3	0.699 [0.557, 0.876]		0.706 [0.560, 0.873]		0.713 [0.561, 0.882]	0.707 [0.563, 0.871]
4	1.081 [0.768, 1.481]		1.072 [0.764, 1.491]		1.076 [0.758, 1.474]	1.064 [0.768, 1.470]
5	0.767 [0.602, 0.971]		0.780 [0.596, 0.996]		0.778 [0.589, 0.991]	0.773 [0.592, 0.990]
6	0.486 [0.345, 0.653]		0.485 [0.343, 0.665]		0.486 [0.349, 0.657]	0.483 [0.340, 0.653]

**Table 5:** Stability of modified-iKM in comparison to the original iKM method

						Modified-iKM			Original iKM	
Interval	Lower	Upper	trisk	nrisk		$\widehat {nrisk}$	$\widehat {ncensor}$		$\widehat {nrisk}$	$\widehat {ncensor}$
When 6-interval information is used:										
1	1	19	0	200		200	6		200	6
2	20	43	10	177		177	3		177	3
3	44	63	20	133		133	41		133	41
4	64	87	30	64		64	15		64	17
5	88	117	40	24		24	3		24	2
6	118	132	50	7		7	5		7	7
When 11-interval information is used:										
1	1	10	0	200		200	0		200	0
2	11	19	5	191		191	6		191	6
3	20	32	10	177		177	0		177	-3
4	33	43	15	153		153	3		153	3
5	44	53	20	133		133	20		133	19
6	54	63	25	100		100	21		100	22
7	64	77	30	64		64	3		64	6
8	78	87	35	39		39	11		39	11
9	88	103	40	24		24	0		24	-2
10	104	117	45	12		12	2		11	2
11	118	132	50	7		7	5		7	7

**Fig. 9:**
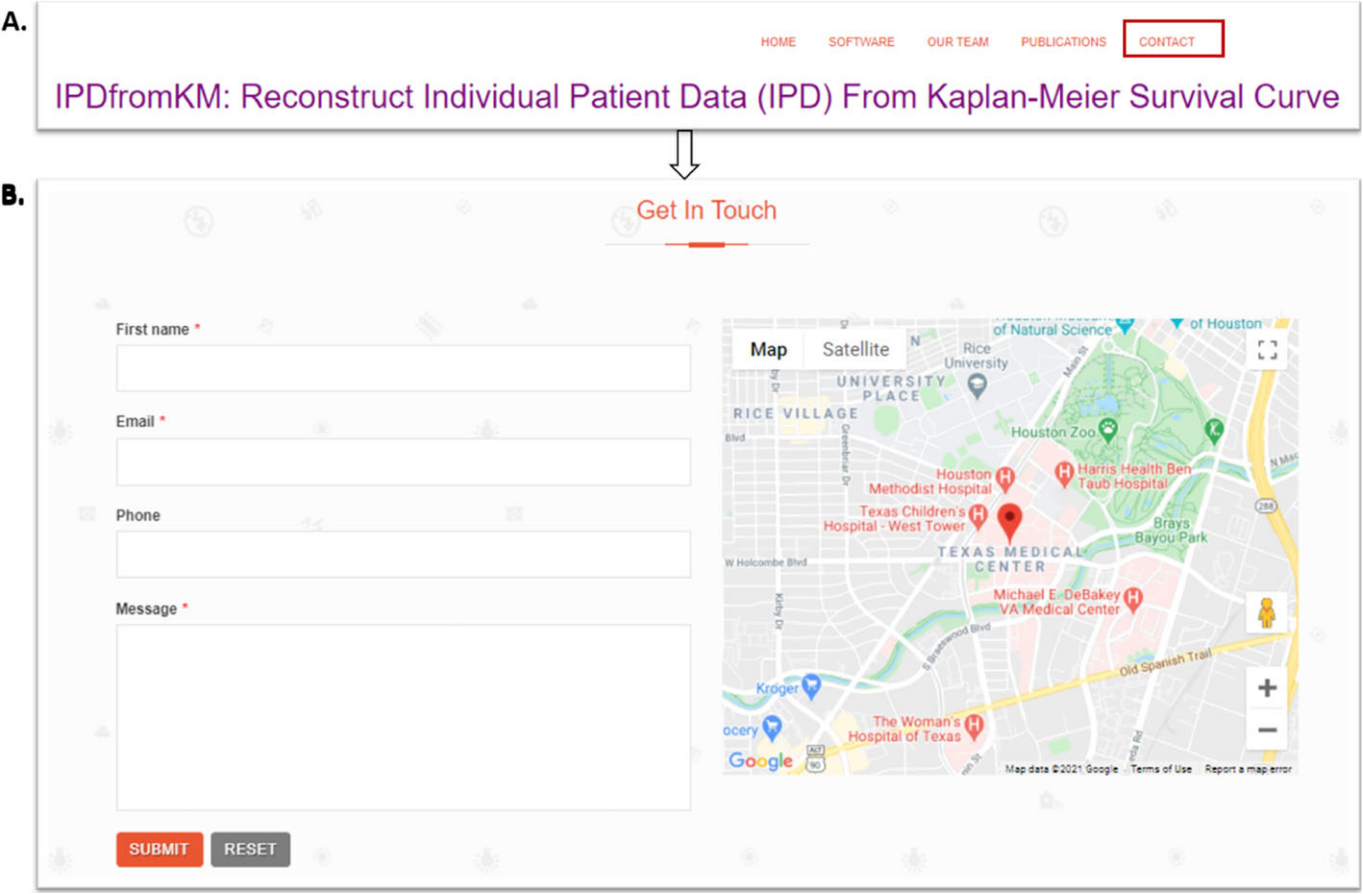
Contact the authors for questions regarding the Shiny application

**Fig. 10:**
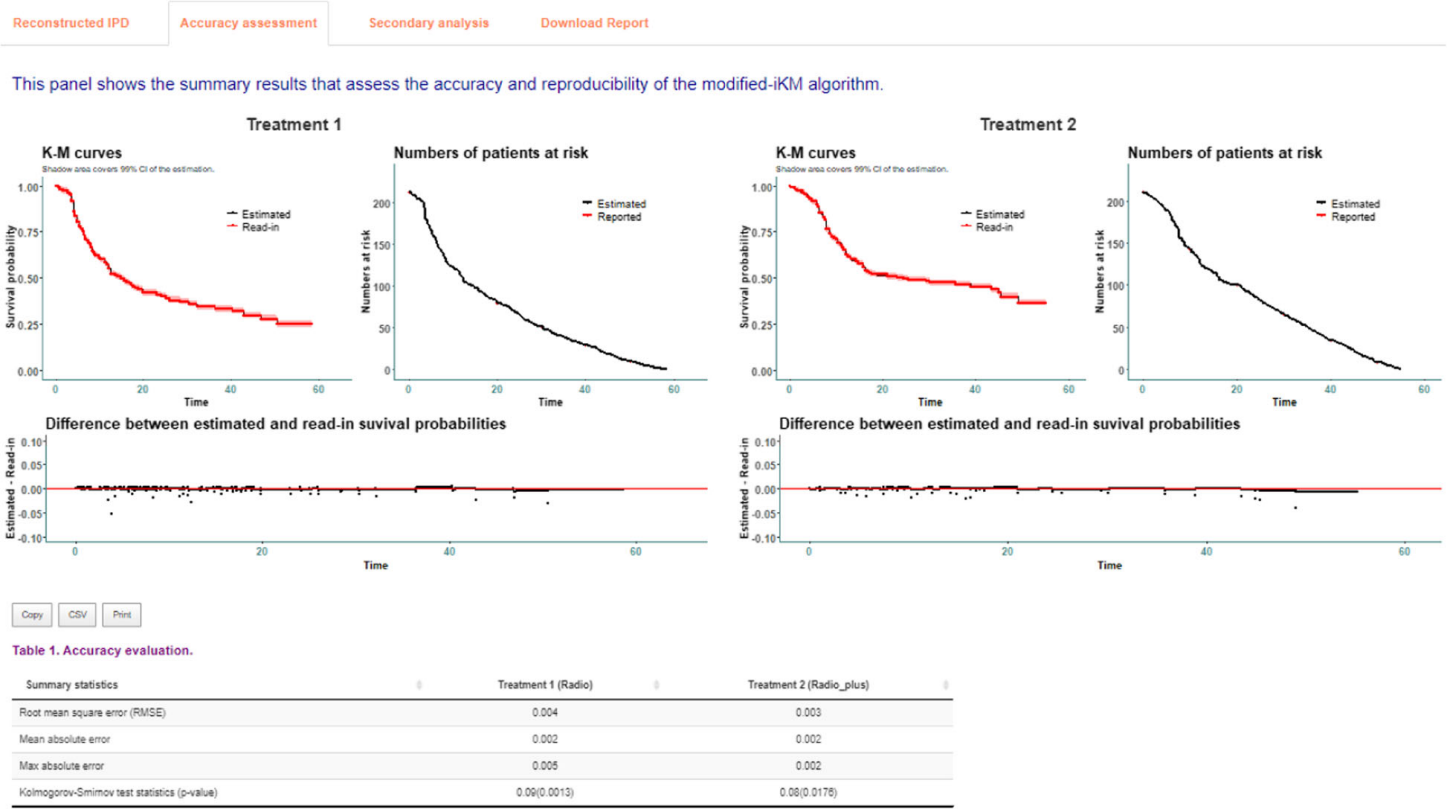
Accuracy assessment using the IPDfromKM Shiny application

**Fig. 11:**
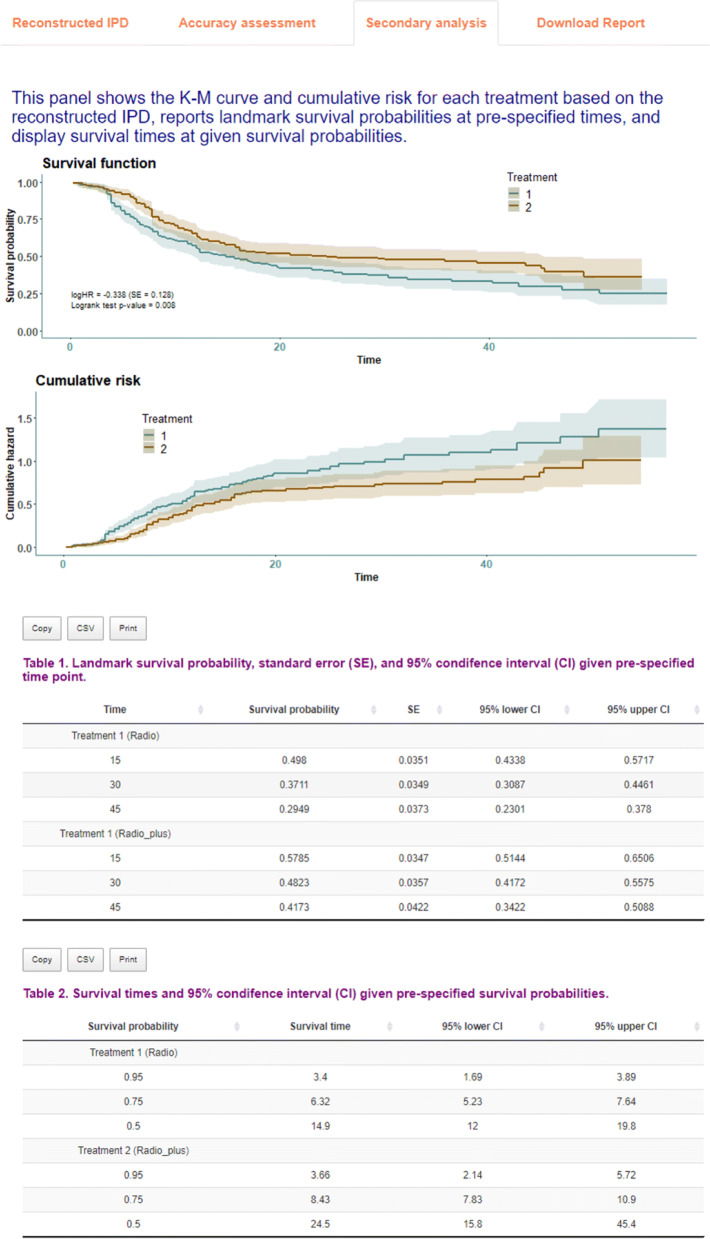
Secondary analysis using the IPDfromKM Shiny application

**Fig. 12:**
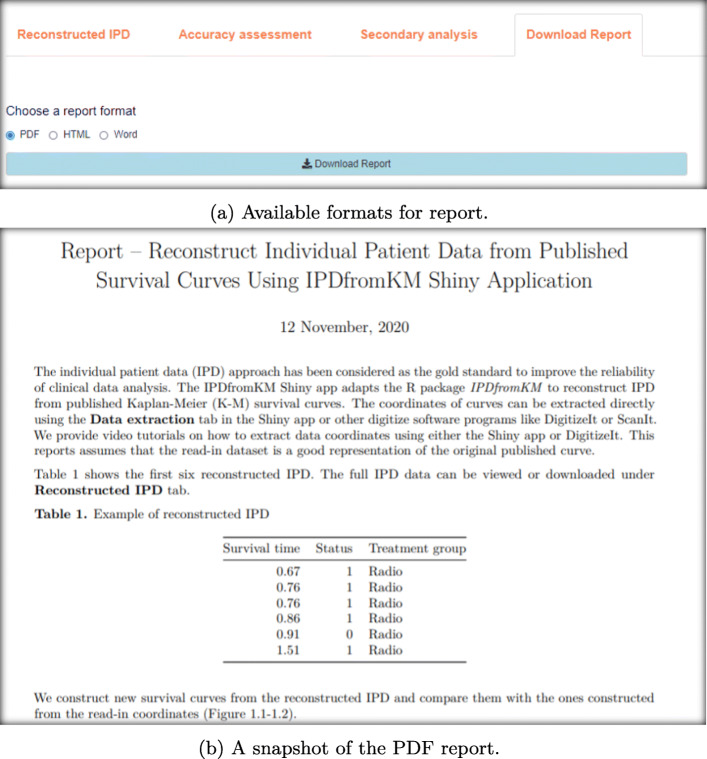
Download report using the IPDfromKM Shiny application

### Figures returned for accuracy assessment or secondary analysis

The *IPDfromKM* package provides the *plot()* function to visualize the accuracy of IPD reconstruction (Fig. 6), in addition to a formal testing. It also provides the *survreport()* function to conduct secondary survival analysis and returns figures for the new analysis (Fig. 7).

### Original K-M curves in the POLAR trial

Figure 8 shows the K-M curves and estimation of median survival and hazard ratio for the subgroups in the POLAR trial.

### Discussion on variability of test statistics

We show in Table 2 that the point estimates for the median OS and HR are close to reported values based on raw data. Considering that the reconstructed IPD data may not be independent and that data extraction may bring up extra variation in the reconstruction process, it is of interest to see how the variability of test statistics based on the reconstructed IPD impacts hypothesis testing. We evaluated this by (1) conducting hypothesis tests using the reconstructed IPD from the five pairs of K-M curves in the POLAR trial and six additional pairs of simulated K-M curves (generated in “Simulation result” section), and (2) constructing bootstrap confidence intervals for the six simulated trials using both true and reconstructed IPDs. Table 3 shows that our method, with reconstructed IPD, reaches the same conclusion as that with the true IPD, indicating that results based on reconstructed IPD are reliable. Table 4 demonstrates that the variability of statistics based on the reconstructed IPD is comparable to that based on the true IPD.

### Long-term commitment of the shiny application

We develop the *IPDfromKM* Shiny application to help users extract data points from published K-M curves and estimate parameters of interest using the modified-iKM. The application is freely available at https://www.trialdesign.org/one-page-shell.html#IPDfromKM and regularly maintained. Figure 9 shows the procedure to contact the authors should users have any questions. Specifically, panel A of Fig. 9 shows the part of the app interface where the “CONTACT” (upper right corner) can be used to communicate with the authors. Upon clicking on “CONTACT,” users will be able to fill out their contact information and specific questions. Responses to users’ questions will be sent back to the users’ email addresses provided in panel B of Fig. 9.

### Additional output of using the app to reconstruct IPD for two treatment groups

## Data Availability

The R code for the project can be download at https://CRAN.R-project.org/package=IPDfromKM. Declarations

## Abbreviations

IPD: Individual patient data
iKM: Iterative algorithm based on Kaplan-Meier estimation
RMSE: Root mean square error
K-M: Kaplan-Meier
